# Associations of apparent temperature with acute cardiac events and subtypes of acute coronary syndromes in Beijing, China

**DOI:** 10.1038/s41598-021-94738-9

**Published:** 2021-07-27

**Authors:** Na Li, Junxiong Ma, Fangjing Liu, Yan Zhang, Pengkun Ma, Yinzi Jin, Zhi-Jie Zheng

**Affiliations:** 1 Department of Global Health, Peking University School of Public Health, 38 Xue Yuan Road, Haidian District, Beijing, 100191 China; 2 Institute for Global Health and Development, Peking University, Beijing, China; 3 Institute of Cardiovascular Disease, Peking University First Hospital, Beijing, China; 4 Institute of Urban Meteorology, Chinese Meteorological Administration, Beijing, China

**Keywords:** Acute coronary syndromes, Epidemiology, Environmental impact

## Abstract

Limited evidence is available on apparent temperature (AT) and hospital admissions for acute cardiac events. We examined the associations of AT with admissions for acute cardiac events and acute coronary syndrome (ACS), and explored the effect difference between ST-elevation myocardial infarction (STEMI) and non-ST-elevation myocardial infarction ACS (NSTE-ACS). Poisson regression with distributed lag non-linear model was applied to examine the temperature-lag-admission associations. Stratified analyses were performed by gender and age-groups for acute cardiac events. A total of 11,657 acute cardiac events admissions were collected from hospital-based chest pain centers in Beijing, during 2017–2019. The single day effect of low AT (− 11 °C, 2.5th percentile) appeared on the 2nd day and persisted until the 11th day, with estimated relative risk (RR) ranging from 1.44 (95% CI: 1.159, 1.790) to 1.084 (95% CI: 1.022, 1.150) for acute cardiac events and from 1.034 (95% CI: 1.010, 1.059) to 1.006 (95% CI: 1.000, 1.011) for ACS. The single day effect of high AT (34 °C, 97.5th percentile) was only observed on the current day. The cold effect on acute cardiac events was more pronounced among female and older patients. The cumulative effect of high AT on STEMI admissions and low AT on NSTE-ACS reached a peak RR peak of 2.545 (95% CI: 1.016, 6.375) and 3.71 (95% CI: 1.315, 10.469) on lag 0–6 days, respectively. Both high and low ATs were associated with increased risk of acute cardiac events and ACS admissions. STEMI admissions may be more sensitive to high AT while NSTE-ACS to low AT.

## Introduction

Ranking the first cause of disability adjusted life years (DALYs) in 50-years-and-older population in 2019, ischemic heart disease (IHD) has been one of the ten most important drivers of increasing disease burden over the past three decades worldwide^1^. In China, IHD ranked the second leading cause of death and approximately 1.7 million deaths were attributable to IHD in 2016^2^. Acute coronary syndrome (ACS) represents an acute manifestation of IHD with a high risk of death, encompassing unstable angina (UA), non-ST-elevation myocardial infarction (NSTEMI), and ST-elevation myocardial infarction (STEMI). According to the latest guidelines, UA and NSTEMI were categorized as non-ST-elevation acute coronary syndromes (NSTE-ACS) because of the similarity in clinical manifestation and management strategies^3,4^. Given the vital importance of immediate medical care for patients with urgent or emergent causes of ACS, it is necessary to identify some modifiable risk factors and take precautionary measures to avoid treatment delays as much as possible, which may help reduce the mortality.

Meteorological factors including ambient temperature, humidity, sunshine, atmospheric pressure, have been reported to be associated with mortality or morbidity of cardiovascular events such as myocardial infarction, out-of-hospital cardiac arrest^5–11^. Extreme temperatures were also linked to increased daily number of hospital admissions, emergency department visits and ambulance dispatches^12–16^. The chest pain is one of the most common chief complaint among the emergency department patients and is associated with potential life-threatening acute cardiac events. Although a number of studies have reported the association between temperature and morbidity of AMI (several were on STEMI), few studies investigated the association stratified by subtypes of AMI. Besides, previous studies indicated an important role for temperature in the incidence of ACS^17^. However, epidemiological studies evaluating the effect of temperature on incidence of ACS are relatively scarce, let alone on effect differences between STEMI and NSTE-ACS^11,18–22^. In view of a threefold increase in the rate of hospitalization for NSTEMI whereas a small reduction for STEMI, improving awareness of the preventive and medical care for NSTE-ACS is important^23^.

In addition, some studies proposed that raw temperature indicator may not accurately describe the true thermal effects, because human perception of ambient temperature depends on both temperature and other meteorological factors such as humidity and wind speed^24,25^. As a combination of meteorological indicators, including ambient temperature, relative humidity and wind velocity, apparent temperature (AT) has been proven to reflect the thermal sensations experienced by the human body more objectively than ambient temperature alone^26^. AT has been adopted to represent the combined exposure of temperature and humidity in several studies exploring the impact of temperature on mental health, behavior and some other diseases^27–29^. However, the study using AT to quantify relationship between temperature and onset of acute cardiac events has not yet been reported.

The aim of our study was to explore the impacts of AT on daily hospital admissions due to acute cardiac events in Beijing, China, from 2017 and 2019. Furthermore, we sought to explore whether the AT-admissions associations varied by gender, age and types of ACS.

## Material and methods

### Study area

Beijing is a typical northern city of China and is located at latitude 39°56′N and longitude 116°20′E, with a permanent population of 21.54 million in 2018 (Beijing Statistical Yearbook 2019 published by China Statistics Press). Beijing has a monsoon influenced humid continental climate with hot humid summers and cold windy dry winters. The annual average temperature ranges from 10 to 12 °C, with average daily minimum temperature of − 6 °C and average daily maximum temperature of 33 °C^28,30^.

### Data collection

Data on admissions due to acute cardiac events in 10 hospital-based chest pain centers in Beijing from March 1st, 2017 to February 28th, 2019 were obtained from the China Chest Pain Center Data Reporting Platform (http://data.chinacpc.org/). All of the ten hospitals passed chest pain center accreditation in 2017 (rather than general hospital) and are designated hospitals for patients with an acute cardiac event in Beijing. Therefore, the data collected from China Chest Pain Center Data Reporting Platform is enough to represent patients with an acute cardiac event during the study period in Beijing. This data reporting platform served as a national surveillance system to evaluate the characteristics, treatments, and outcomes of patients hospitalized with acute cardiac events, launched by the Chinese Cardiovascular Association. To be eligible, we included data from those who meet the following criteria: (1) are aged 18 years or older; (2) are admitted by all kinds of modes including directly by self, via emergency medical services, transferred-in or in-hospital; (3) have a primary diagnosis of cardiogenic chest pain, including the STEMI, NSTEMI, UA, aortic dissection, pulmonary embolism, and others.

We derived meteorological data from the China Meteorological Data Sharing Service System (http://data.cma.cn/). Air pollution data including carbon monoxide (CO), nitrogen dioxide (NO_2_), particulate matter with aerodynamic diameter less than 10 µm (PM_10_), particulate matter with aerodynamic diameter less than 2.5 µm (PM_2.5_), sulfur dioxide (SO_2_) and ozone (O_3_) was collected from Beijing Municipal Ecological and Environmental Monitoring Center (http://www.bjmemc.com.cn/). We averaged the daily mean concentrations of air pollutants from the available monitoring data across seven national air monitoring stations evenly distributed in urban districts of Beijing. Extreme low and high daily temperatures are defined as those daily mean temperatures that fall below the 2.5th percentile and above the 97.5th percentile of the total daily apparent temperature distribution, respectively^31,32^. Using this percentile-based definition, the extreme low temperature threshold is less than or equal to − 11 °C, and the high extreme threshold is greater than or equal to 34 °C for the period studied.

### Calculation of AT

The AT was calculated by the common meteorological indicators, including daily mean temperature, relative humidity, wind velocity and water vapor pressure using the following equations^25,28,33^:

1 $$AT = T + 0.33*e - 0.70*WS - 4.00$$

2 $$e = RH/100*6.105*\exp \left[ {17.27*T/\left( {237.7 + T} \right)} \right]$$

In Eq. (1), T, *e* and WS denote the daily mean temperature (°C), water vapor pressure (hPa) and average wind velocity (m/s), respectively. The water vapor pressure *e* is calculated from Eq. (2) using the daily mean temperature and relative humidity (RH, %).

### Statistic analysis

Firstly, a descriptive analysis was conducted for daily hospital admissions, meteorological variables and air pollutants datasets. Then, time series analyses were conducted to investigate the effects of exposure to extreme apparent temperature (low and high) on daily hospital admissions.

A generalized linear model (GLM) approach with a quasi-likelihood Poisson distribution for dispersion data was conducted to investigate the association between daily hospital admissions and extreme temperature conditions (both low and high). A natural cubic B-spline with seven degrees of freedom per year was used to control for the long-term trend and seasonal cycles^32,34^. The model included the following confounding factors: day of week (DOW), public holidays (Holiday), PM_10_ and O_3_. The lagged and potential non-linear effect of AT were evaluated by distributed lag non-linear model (DLNM)^19,27,31,35^ by adding a cross-basis function with up to 14 lag days, using a quadratic B-spline with three internal knots at the 10th, 75th, and 90th percentiles location of AT distributions and three knots in log response^34,36^.

A new metric Minimum Admission Apparent Temperature (MAAT) at minimum relative risk was defined in this study, which is similar to Minimum Mortality Temperature (MMT) defined by previous studies^14,32^. MAAT represents the minimum of the exposure–response or the temperature at the lowest risk obtained from specific model. Thus, the cold and heat effects were defined as the hospital admission risks at the 2.5th and 97.5th temperature percentiles, compared with the minimum-hospital admission temperature percentile. The approach is well documented^37,38^ and has been used to estimate the impact of temperature on mortality. Stratified analyses were performed by gender and age groups for acute cardiac events admissions. And the analyses for different diagnostic types of diseases (ACS, STEMI and NSTE-ACS) were also conducted through the above steps. The RR for each category was compared to the reference MAAT and reported with both 95% confidence intervals (CI). All statistical analyses were carried out in R software (version 3.6.3) using “*dlnm*” package^38^.

### Ethical approval

Ethical approval for the study was obtained from the institutional review boards of the GUSU group ethics committee (GUSU19005), established by Chinese Cardiovascular Association. The need for informed consent was waived by the Institutional Review Board because the health information was primarily the hospital specific daily count of admissions, i.e., summarized data (overall, and by age and sex subgroups) without any individual identifiers. The study was performed in accordance with the Declaration of Helsinki.

## Results

### Descriptive statistics of daily admissions and meteorological variables

**Figure 1:**
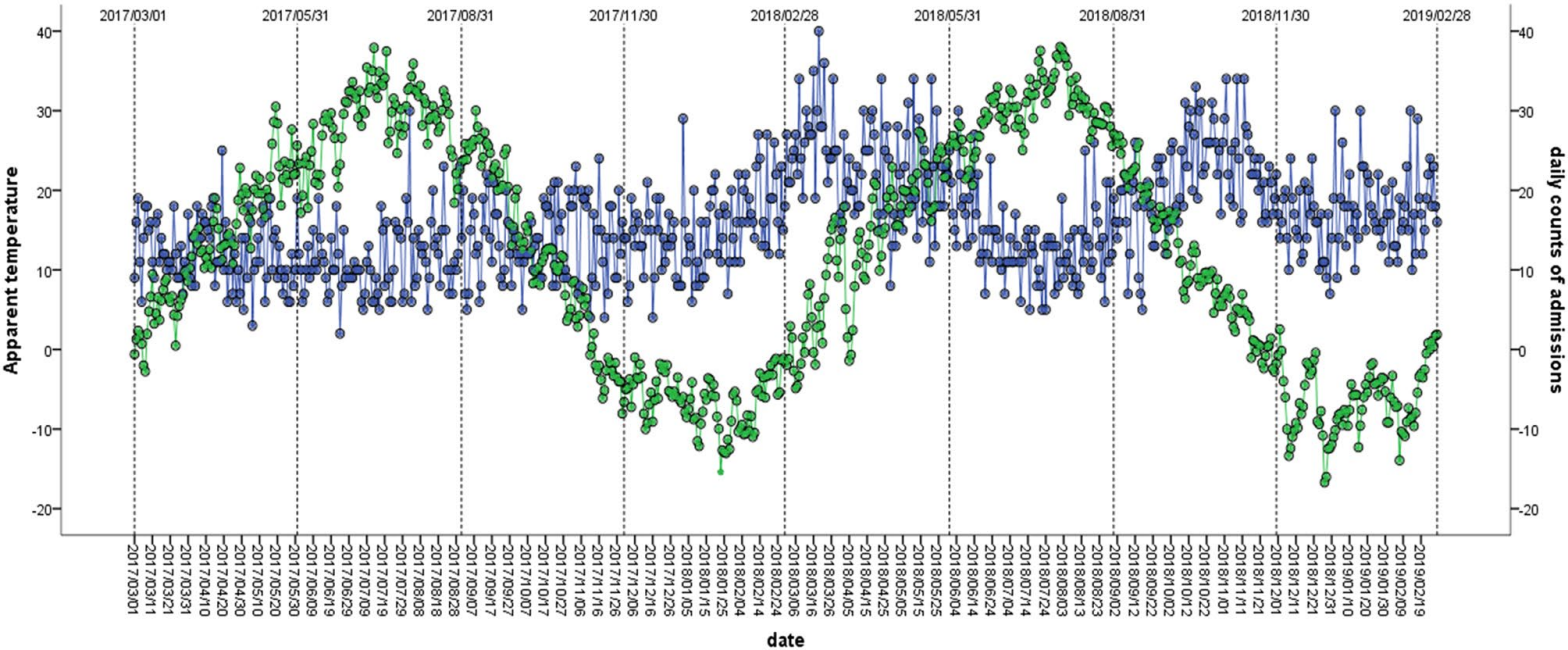
Seasonal distribution of daily hospital admissions due to acute cardiac events and apparent temperature in 10 hospitals in Beijing between 1st March 2017 and 28th February 2019. *Green points indicate apparent temperature; blue points indicate daily admission for acute cardiac events.

We included a total of 14,447 chest pain admissions (11,657 admissions were due to acute cardiac events) in 10 public hospitals in Beijing between March, 1st 2017 to February, 28th 2019 (730 days). Among these acute cardiac events admissions, male and patients aged above 65 years accounted for 65.1% and 42.3%, respectively (Table 1). The descriptive statistics for the meteorological and air pollution parameters were summarized in Table 2. Daily average AT was 11.9 °C, slightly lower than mean ambient temperature (13.8 °C). The time-series distribution of daily admissions due to acute cardiac events exhibited a seasonal pattern, with more hospital admissions due to acute cardiac events in lower temperature days (Fig. 1).

**Table 1:** Summary statistics of daily hospital admissions between 1st March 2017 and 28th February 2019.

Category	Sum	Mean ± SD	Percentiles				
			Min	P_25_	P_50_	P_75_	Max
Chest pain	14,447	19.8 ± 8.9	2	13	19	26	55
Acute cardiac events	11,657	15.8 ± 6.5	2	11	15	20	40
Female	4072	5.6 ± 3.4	0	3	5	8	20
Male	7582	10.4 ± 4.3	1	7	10	10	26
< 65 years	6725	9.2 ± 4.2	1	6	9	12	24
≥ 65 years	4931	6.8 ± 3.5	0	4	6	9	19
ACS	8006	11.0 ± 4.9	0	7	10	14	28
STEMI	3161	4.3 ± 2.6	0	3	4	6	18
NSTE-ACS	4845	6.6 ± 4.4	0	3	6	9	21

ACS, acute coronary syndrome; STEMI, ST-segment-elevation myocardial infarction; NSTE-ACS, Non-STEMI acute coronary syndrome.

**Table 2:** Summary statistics of daily meteorological and air pollution data between 1st March 2017 and 28th February 2019.

Environmental variables	Mean ± SD	Percentiles				
		Min	P_25_	P_50_	P_75_	Max
AT	11.90 ± 14.38	−16.7	−1.9	12.5	25.1	38.01
T_mean_	13.80 ± 11.69	−9.2	2.6	15.3	24.5	32.5
Precipitation	1.53 ± 7.42	0.0	0.0	0.0	0.0	86.2
BP	1013.18 ± 10.37	991.9	1004.0	1013.0	1021.0	1040.0
WS	2.03 ± 0.82	0.5	1.5	1.9	2.4	6.2
RH	48.51 ± 19.96	11.0	31.5	47	65	93.5
CO	0.81 ± 0.41	0.2	0.5	0.8	1.0	2.9
NO_2_	40.72 ± 18.02	6.7	27.3	37.4	51.3	104.4
SO_2_	6.10 ± 4.31	2.0	2.9	4.7	7.9	32.4
O_3_	60.99 ± 36.96	3.6	34.3	54.1	81.4	182.5
PM_2.5_	50.46 ± 41.50	3.9	21.4	39.9	67.0	343.0
PM_10_	82.99 ± 64.66	9.0	44.3	67.7	104.3	883.3
AQI	80.82 ± 49.60	13.9	46.5	68.2	102.4	425.1

SD, standard deviation; AT, apparent temperature; T_mean_, daily mean of ambient temperature; BP, barometric pressure; WS, wind speed; RH, relative humidity; CO, carbon monoxide; NO_2_, nitrogen dioxide; SO_2_, sulfur dioxide; O_3_, ozone; PM_2.5_, particulate matter with a diameter less than 2.5 µm; PM_10_, particulate matter with a diameter ≥ 10 µm; AQI, air quality index.

### The impact of extreme low and high AT on daily admissions due to acute cardiac events

**Figure 2:**
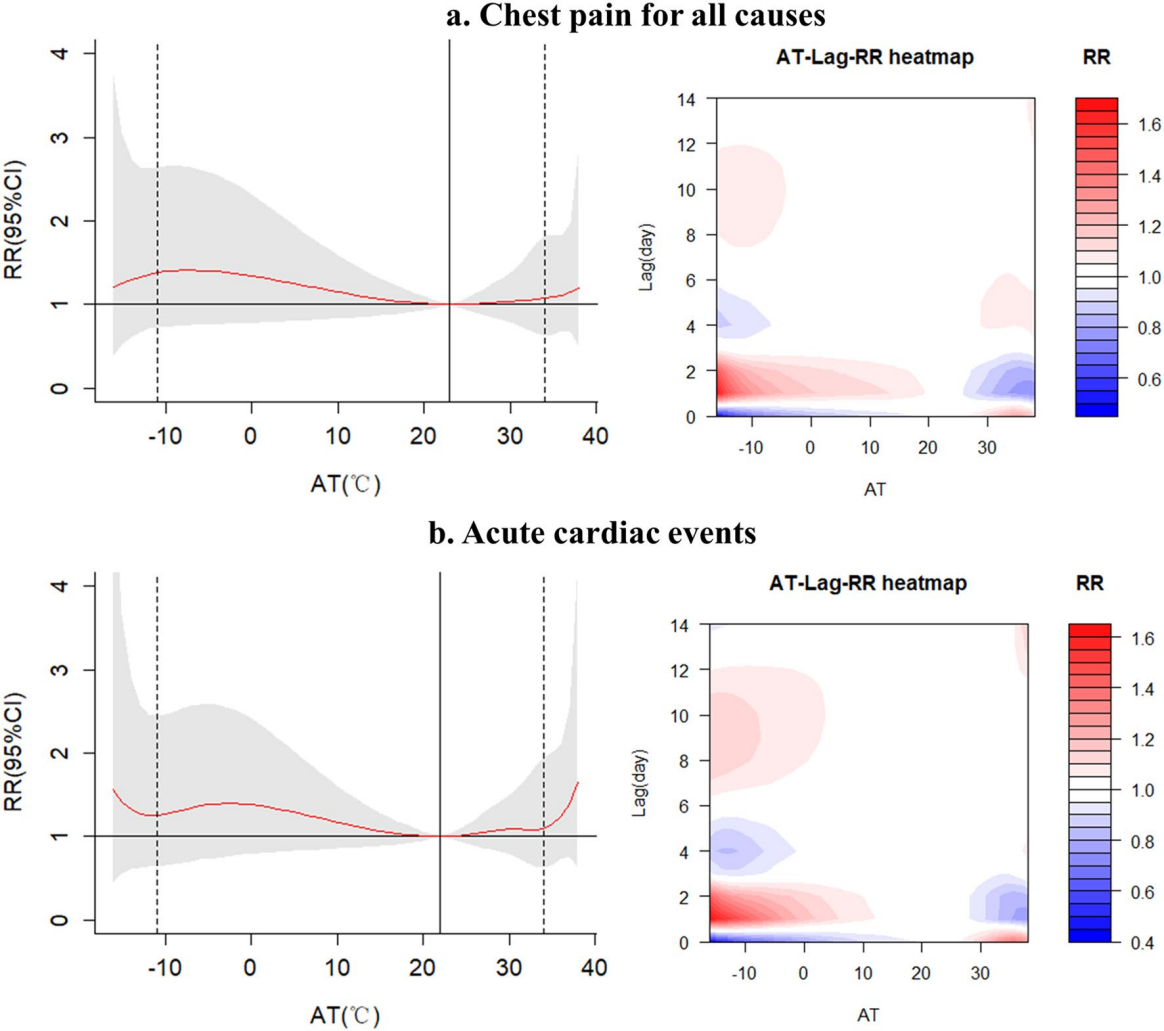
Exposure–response relationship between apparent temperature (AT) and daily hospital admissions due to chest pain in 10 public hospitals in Beijing, China (with reference of the Minimum Admission Apparent Temperature, MAAT).

**Figure 3:**
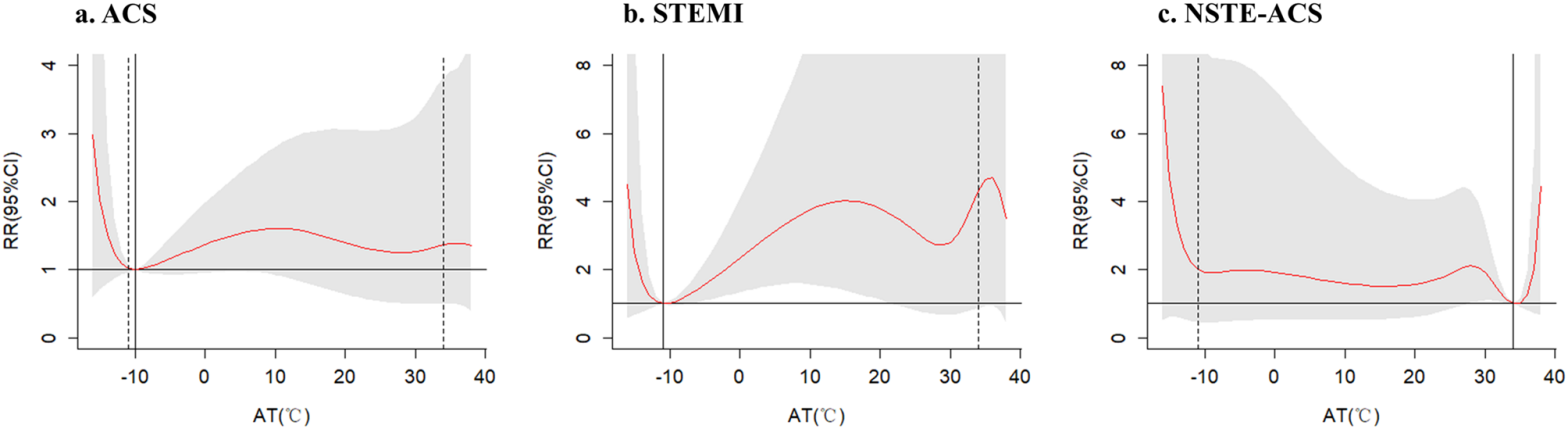
Exposure–response relationship between apparent temperature (AT) and daily hospital admissions due to acute coronary syndrome (ACS) in disease-specific groups (with reference of the Minimum Admission Apparent Temperature, MAAT). Abbreviations: ACS, acute coronary syndrome; STEMI, ST-segment-elevated myocardial infarction; NSTE-ACS, Non-STEMI acute coronary syndrome. Notes: MAAT for ACS, STEMI and NSTE-ACS was − 10 °C, − 11 °C and 34 °C, respectively.

The exposure–response curves between AT and daily admissions due to chest pain were shown in Fig. 2, with MAAT of 23.0 °C (70.8th percentile) for all cause-chest pain and 22.0 °C (69.0th percentile) for acute cardiac events and, respectively. Both all cause-chest pain and acute cardiac events show more of a U type curve. Figure 3 showed non-linear relationships between AT and daily admissions for subtypes of ACS. The MAAT for STEMI and NSTE-ACS were found to be − 11 °C and 34 °C, respectively, suggesting that the extreme high AT tended to have a larger effect on admissions caused by STEMI while low extremes tended to have a greater effect on NSTE-ACS admissions.

The single lag effect of low AT and high AT on subtype causes of chest pain were presented in Table 3. Both extreme low and high ATs were associated with increased risks (RR > 1) for all-cause chest pain, acute cardiac events and ACS compared with MAAT, whereas admissions caused by NSTE-ACS were only associated with increased risks at extreme high AT. The MAATs for chest pain admissions caused by STEMI and NSTE-ACS were − 11 °C and 34 °C, corresponding to the extreme low AT and extreme high AT in this study. Hence, we did not exhibit the results of cold effect and heat effect for them (RR = 1).

**Table 3:** The single day lag effects of extreme apparent temperature at different lag day(s) with the reference of the Minimum Admission Apparent Temperature (MAAT), by different chest pain categories.

	All-cause chest pain		Acute cardiac events		ACS		NSTE-ACS	STEMI
	Cold effect	Heat effect	Cold effect	Heat effect	Cold effect	Heat effect	Cold effect	Heat effect
MAAT, °C (percentile)	23 (70.8)		22 (69)		−10 (4.3)		34 (97.5)	−11 (2.5)
lag0	0.68^a^ (0.484, 0.955)	1.266 (0.987, 1.623)	0.624 (0.437, 0.891)	1.352^a^ (1.03, 1.775)	0.969 (0.938, 1.001)	1.985^a^ (1.124, 3.506)	1.112 (0.52, 2.379)	5.223^a^ (2.143, 12.727)
lag1	1.336^a^ (1.088, 1.640)	0.792^a^ (0.676, 0.927)	1.440^a^ (1.159, 1.790)	0.841^a^ (0.709, 0.998)	1.034^a^ (1.010, 1.059)	0.666^a^ (0.476, 0.933)	1.511 (0.958, 2.382)	0.633 (0.373, 1.075)
lag2	1.218^a^ (1.053, 1.407)	0.838^a^ (0.749, 0.937)	1.236^a^ (1.060, 1.440)	0.835^a^ (0.74, 0.943)	1.018^a^ (1.002, 1.035)	0.712^a^ (0.56, 0.904)	1.333 (0.968, 1.835)	0.675^a^ (0.462, 0.985)
lag3	0.977 (0.886, 1.079)	0.983 (0.903, 1.07)	0.919 (0.828, 1.02)	0.934 (0.852, 1.024)	0.993 (0.982, 1.003)	0.965 (0.814, 1.142)	1.107 (0.882, 1.391)	1.105 (0.849, 1.439)
lag4	0.918 (0.824, 1.022)	1.053 (0.963, 1.152)	0.856 (0.764, 0.96)	0.992 (0.900, 1.093)	0.987 (0.975, 0.999)	1.084 (0.903, 1.301)	1.019 (0.795, 1.307)	1.26 (0.949, 1.673)
lag5	0.945 (0.872, 1.025)	1.065 (0.997, 1.138)	0.909 (0.834, 0.989)	1.017 (0.946, 1.093)	0.993 (0.984, 1.001)	1.087 (0.949, 1.246)	1.001 (0.831, 1.204)	1.165 (0.945, 1.437)
lag6	0.999 (0.94, 1.061)	1.054^a^ (1.003, 1.107)	0.995 (0.934, 1.06)	1.027 (0.973, 1.084)	1.001 (0.995, 1.007)	1.05 (0.949, 1.163)	1.006 (0.877, 1.154)	1.024 (0.877, 1.196)
lag7	1.039 (0.974, 1.109)	1.042 (0.989, 1.098)	1.061 (0.991, 1.135)	1.032 (0.975, 1.092)	1.006^a^ (1, 1.013)	1.024 (0.919, 1.14)	1.011 (0.875, 1.169)	0.942 (0.798, 1.111)
lag8	1.065 (0.992, 1.142)	1.032 (0.975, 1.092)	1.100^a^ (1.022, 1.184)	1.034 (0.972, 1.099)	1.009^a^ (1.002, 1.017)	1.008 (0.897, 1.133)	1.015 (0.867, 1.187)	0.906 (0.757, 1.085)
lag9	1.076^a^ (1.004, 1.153)	1.023 (0.968, 1.081)	1.115^a^ (1.037, 1.198)	1.033 (0.973, 1.096)	1.010^a^ (1.003, 1.017)	1.002 (0.895, 1.123)	1.017 (0.873, 1.185)	0.906 (0.76, 1.08)
lag10	1.076^a^ (1.011, 1.144)	1.014 (0.966, 1.065)	1.108^a^ (1.038, 1.182)	1.029 (0.976, 1.085)	1.009^a^ (1.002, 1.015)	1.004 (0.907, 1.111)	1.018 (0.888, 1.167)	0.935 (0.8, 1.094)
lag11	1.065^a^ (1.007, 1.128)	1.007 (0.962, 1.053)	1.084^a^ (1.022, 1.15)	1.024 (0.975, 1.075)	1.006^a^ (1, 1.011)	1.012 (0.923, 1.11)	1.018 (0.9, 1.152)	0.991 (0.86, 1.143)
lag12	1.048 (0.983, 1.118)	1 (0.948, 1.054)	1.048 (0.98, 1.121)	1.017 (0.961, 1.077)	1.002 (0.996, 1.008)	1.025 (0.923, 1.138)	1.017 (0.884, 1.17)	1.071 (0.909, 1.262)
lag13	1.027 (0.94, 1.122)	0.993 (0.923, 1.069)	1.006 (0.917, 1.103)	1.01 (0.933, 1.093)	0.997 (0.989, 1.006)	1.041 (0.901, 1.202)	1.016 (0.837, 1.232)	1.173 (0.934, 1.472)
lag14	1.004 (0.889, 1.133)	0.986 (0.892, 1.091)	0.961 (0.847, 1.092)	1.002 (0.899, 1.117)	0.992 (0.98, 1.005)	1.058 (0.868, 1.291)	1.014 (0.777, 1.325)	1.292 (0.944, 1.77)

ACS, Acute coronary syndrome; STEMI, ST-segment-elevation myocardial infarction; NSTE-ACS, Non-STEMI acute coronary syndrome.

^a^Relative risk values are statistically significant.

The single day effects of extreme low AT on admissions for all-cause chest pain, acute cardiac events and ACS could be observed from the 2nd day to the 11th day of exposure, but not continuously (Table 3). The highest risk associated with low AT for all three causes emerged on the 2nd day. While for extreme high AT, the single day effect was only observed on the 6th lag day for all-cause chest pain, and on the 1st lag day for acute cardiac events and ACS. For example, the risk of admission due to all-cause chest pain, acute cardiac events and ACS admission increased by 33.6% (RR = 1.336, 95% CI: 1.088–1.640), 44% (RR = 1.440, 95% CI: 1.159–1.790) and 3.4% (RR = 1.034, 95% CI: 1.010–1.059), respectively at the extreme low AT.

Table 4 provides the cumulative effect of exposure to extreme ATs compared with the reference. The cumulative effects of extreme high AT on STEMI admissions could be observed on the day of exposure until the 8th day, with RR varying from 3.308 (95% CI: 1.454, 7.526) at lag 0–1 days to 3.165 (95% CI: 1.015, 9.868) at lag 0–8 days. While for NSTE-ACS, the cumulative effects of extreme low AT could be observed on the 2nd day after exposure until the 7th day with RRs varying from 2.240 (95% CI: 1.086, 4.621) at lag 0–2 days of to 2.545 (95% CI: 1.016–6.375) at lag 0–6 days. However, we did not observe significant cumulative effects of extreme ATs for all-cause chest pain, acute cardiac events and ACS.

**Table 4:** The cumulative effect estimates of extreme apparent temperature at different lag day(s) with the reference of the Minimum Admission Apparent Temperature (MAAT), by different chest pain categories.

	All-cause chest pain		Acute cardiac events		ACS		NSTE-ACS	STEMI
	Cold effect	Heat effect	Cold effect	Heat effect	Cold effect	Heat effect	Cold effect	Heat effect
MAAT, °C (percentile)	23 (70.8)		22 (69)		−10 (4.3)		34 (97.5)	−11 (2.5)
lag0-1	0.908 (0.666, 1.238)	1.002 (0.79, 1.272)	0.898 (0.65, 1.241)	1.137 (0.877, 1.476)	1.002 (0.973, 1.031)	1.322 (0.779, 2.244)	1.680 (0.826, 3.417)	3.308^a^ (1.454, 7.526)
lag0-2	1.105 (0.803, 1.521)	0.84 (0.653, 1.08)	1.11 (0.796, 1.547)	0.95 (0.723, 1.249)	1.02 (0.989, 1.053)	0.941 (0.55, 1.612)	2.240^a^ (1.086, 4.621)	2.231^a^ (0.968, 5.14)
lag0-3	1.08 (0.78, 1.496)	0.826 (0.638, 1.069)	1.021 (0.727, 1.432)	0.887 (0.67, 1.175)	1.013 (0.983, 1.043)	0.908 (0.527, 1.564)	2.481^a^ (1.196, 5.145)	2.466^a^ (1.063, 5.718)
lag0-4	0.991 (0.699, 1.406)	0.869 (0.658, 1.15)	0.874 (0.607, 1.257)	0.88 (0.649, 1.192)	1 (0.97, 1.031)	0.984 (0.55, 1.761)	2.529^a^ (1.159, 5.518)	3.108^a^ (1.269, 7.614)
lag0-5	0.937 (0.639, 1.375)	0.926 (0.682, 1.258)	0.794 (0.532, 1.184)	0.895 (0.641, 1.25)	0.992 (0.96, 1.026)	1.07 (0.566, 2.025)	2.530^a^ (1.073, 5.967)	3.621^a^ (1.362, 9.625)
lag0-6	0.936 (0.622, 1.408)	0.975 (0.704, 1.351)	0.79 (0.516, 1.21)	0.919 (0.644, 1.311)	0.993 (0.958, 1.03)	1.124 (0.569, 2.219)	2.545^a^ (1.016, 6.375)	3.71 (1.315, 10.469)
lag0-7	0.973 (0.633, 1.494)	1.016 (0.723, 1.429)	0.838 (0.535, 1.31)	0.948 (0.654, 1.375)	0.999 (0.962, 1.038)	1.151 (0.563, 2.349)	2.574 (0.978, 6.77)	3.494^a^ (1.181, 10.332)
lag0-8	1.036 (0.659, 1.628)	1.049 (0.733, 1.5)	0.922 (0.575, 1.476)	0.98 (0.664, 1.448)	1.009 (0.968, 1.05)	1.16 (0.547, 2.459)	2.612 (0.941, 7.246)	3.165^a^ (1.015, 9.868)
lag0-9	1.115 (0.689, 1.802)	1.072 (0.734, 1.567)	1.027 (0.623, 1.693)	1.012 (0.669, 1.531)	1.019 (0.975, 1.064)	1.163 (0.524, 2.578)	2.656 (0.899, 7.843)	2.867 (0.861, 9.544)
lag0-10	1.199 (0.719, 1.999)	1.088 (0.726, 1.63)	1.138 (0.669, 1.936)	1.042 (0.671, 1.618)	1.027 (0.981, 1.076)	1.168 (0.502, 2.717)	2.704 (0.857, 8.532)	2.682 (0.749, 9.596)
lag0-11	1.277 (0.744, 2.194)	1.095 (0.713, 1.682)	1.234 (0.704, 2.163)	1.067 (0.669, 1.701)	1.033 (0.984, 1.086)	1.182 (0.485, 2.879)	2.752 (0.819, 9.245)	2.659 (0.693, 10.195)
lag0-12	1.339 (0.758, 2.366)	1.095 (0.695, 1.725)	1.293 (0.717, 2.333)	1.085 (0.663, 1.777)	1.035 (0.984, 1.09)	1.211 (0.476, 3.079)	2.799 (0.787, 9.961)	2.848 (0.696, 11.659)
lag0-13	1.375 (0.753, 2.51)	1.087 (0.669, 1.766)	1.301 (0.698, 2.425)	1.096 (0.649, 1.852)	1.033 (0.979, 1.089)	1.26 (0.472, 3.364)	2.843 (0.749, 10.788)	3.341 (0.757, 14.751)
lag0-14	1.38 (0.721, 2.642)	1.072 (0.631, 1.822)	1.25 (0.638, 2.449)	1.098 (0.62, 1.945)	1.025 (0.968, 1.084)	1.334 (0.464, 3.832)	2.884 (0.69, 12.05)	4.317 (0.868, 21.455)

ACS, Acute coronary syndrome; STEMI, ST-segment elevation myocardial infarction; NSTE-ACS, Non-STEMI acute coronary syndrome.

^a^Relative risk values are statistically significant.

**Figure 4:**
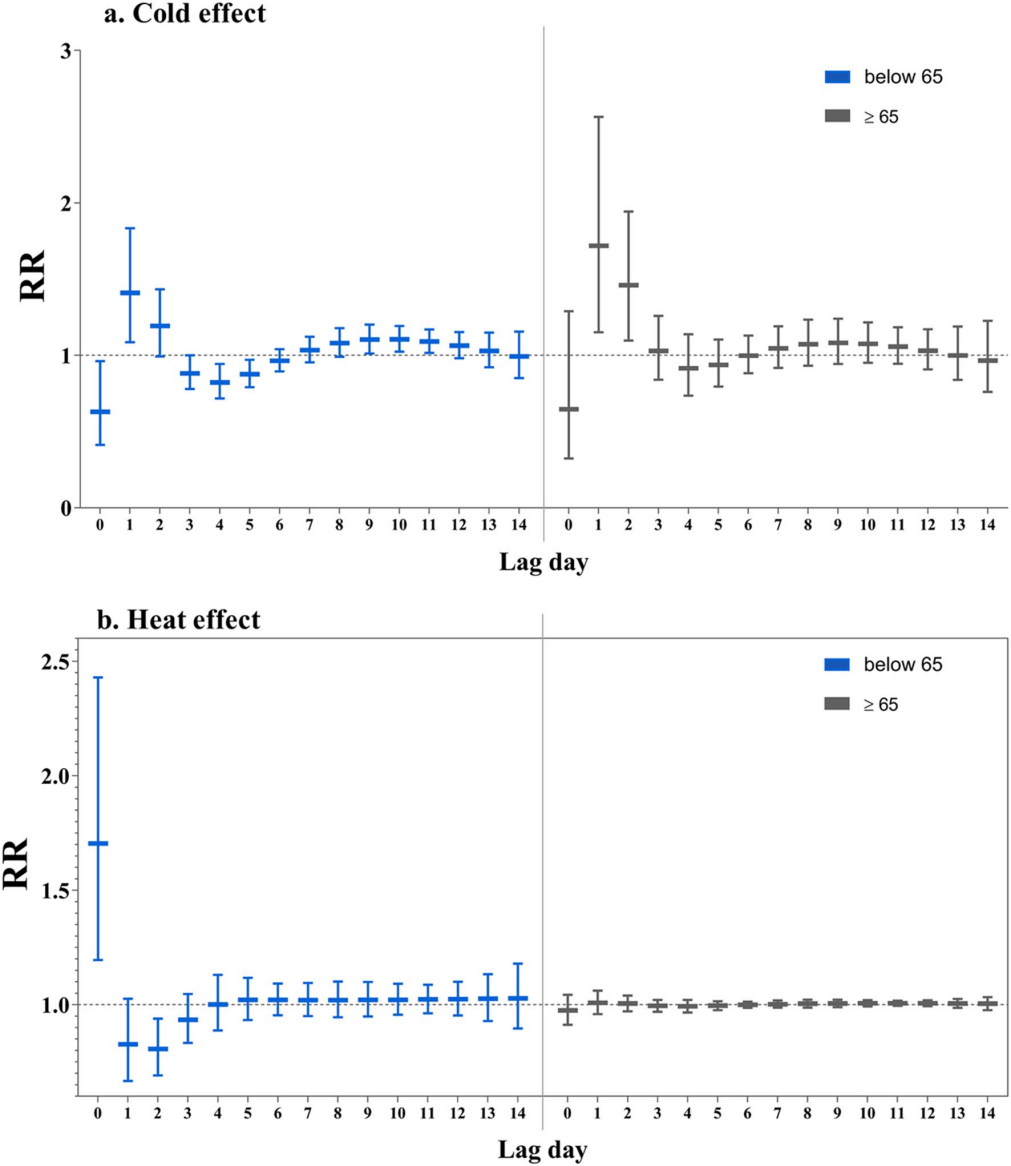
The single day effects of high AT for admissions due to acute cardiac events cross different lag day(s) in age-specific groups, with the reference of the Minimum Admission Apparent Temperature (MAAT) of each group. Notes: MAAT for < 65 group and ≥ 65 group was 20 °C and 33 °C, respectively.

**Figure 5:**
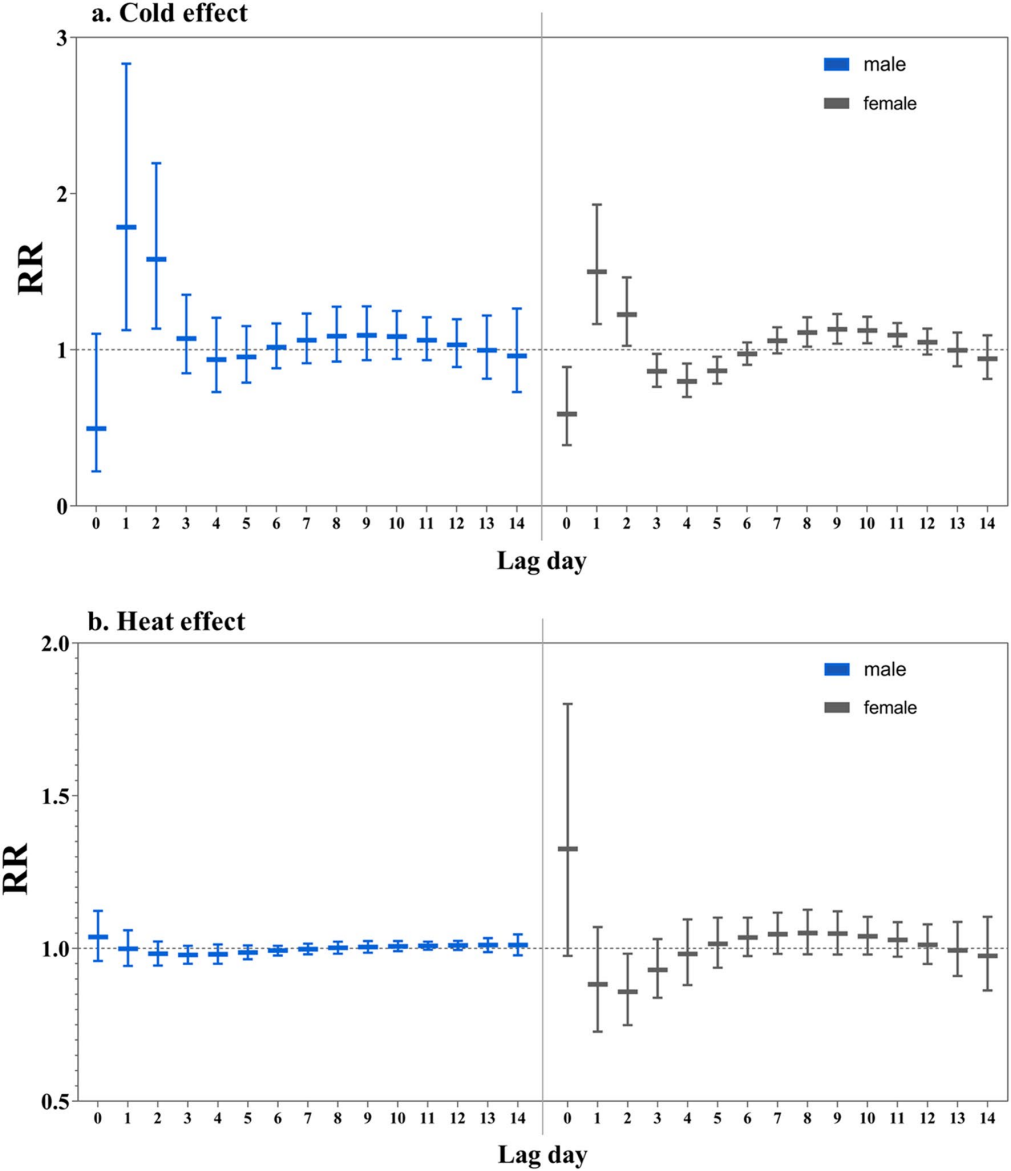
The single day effects of extreme apparent temperature for admissions due to acute cardiac events cross different lag day(s) in gender-specific groups, with the reference of the Minimum Admission Apparent Temperature (MAAT) of each group. Notes: MAAT for male and female group was 33 °C and 22 °C, respectively.

The effects of extreme low AT seemed to be more evident among the female and the patients aged over 65 years, compared with the male and the population aged below 65 years, respectively (Figs. 4, 5, Tables S1, S2). For instance, the maximum RR of cold effect were 1.718 (95% CI: 1.151, 2.564) for patients aged over 65 years at the 2nd lag day while 1.410 (95% CI: 1.085, 1.833) for patients aged below 65 years. The highest RR was 1.785 (95% CI: 1.126, 2.831) for the female and 1.499 (95% CI: 1.165, 1.930) for the male. However, the cold effect for patients below 65 years and the male were longer lasting and could still be observed at lag 8–11 days. With respect to heat effect, the highest RR was observed for patients below 65 on the current day of exposure (RR = 1.704, 95% CI: 1.195, 2.429).

## Discussion

Our findings support non-linear relationships between AT and chest pain admissions caused by ACS (including STEMI and NSTE-ACS), such that the risk of acute cardiac events onset is higher at both extreme high and low ATs relative to the MAAT. The effect of low AT on acute cardiac event admissions appeared one day after the exposure and can still be observed on the 8th and 11th days after the exposure whereas the effect of high AT was immediate and relatively short-lived. In addition, the current study found that MAAT of STEMI is close to extreme low temperatures, indicating increased risks at high temperature. Conversely, the minimum risk temperature of NSTE-ACS is close to extreme high temperatures, indicating that extreme low AT tend to have significant effect on NSTE-ACS rather than high AT.

Overall, our findings contribute to the limited epidemiological evidence for the adverse effects of non-optimum extreme temperatures on the onset of acute cardiac events and incidence of ACS in the area of subtropical monsoon climate, and may be of major population and clinical implications. We adopted the new comprehensive index of AT as a potential warning index, which may be a more realistic and objective measure than ambient temperature alone. The associations of extreme ATs with acute cardiac events admissions may offer implications regarding mobilization and improved utilization of public and emergency resources in the city. For example, in high- or low-temperature weather conditions, public service announcement or ads and banners could be used to alert the public. Similarly, emergency medical system and emergency medical staff could also be alerted for a possible surge in calls and services. Adaptation strategies such as using air conditioners in summer and adequate hydration should be considered. Although the risk of climate and environmental triggers to any single individual is relatively small compared with the effect of well-documented risk factors (e.g. smoking), the public health relevance is considerable, and small percentage increases can translate into substantial absolute increases, because the incidence of chest pain are common and exposure to temperature can be ubiquitous.

Our findings are consistent with several studies reporting the impact of extreme temperature on admissions due to ACS or chest pain. A study exploring impact of extreme temperatures on ambulance dispatches in London reported that the minimum ambulance dispatch temperature (TMADT) for chest pain was less than the 1st temperature percentile and found increased risks only at higher temperature. Whereas the TMADT for acute cardiac events was above the 99th percentile indicating increased risks only at higher temperatures^14^. Our study showed that the TMAAT for all-cause chest pain (23 °C) and acute cardiac events (22 °C) admissions were close to each other. We found significant increased risks for both at extreme low and high ATs, and the RRs for acute cardiac events were larger than those for all-cause chest pain. In addition, the heat effect for acute cardiac events could be observed on the current day while it could not be observed until on the 6th day after exposure for all-cause chest pain. This inconsistency in research findings could be due to the major difference in dataset used, study population, analytical method as well as temporal and spatial properties.

Our results did not find cumulative effects of extreme ATs on acute cardiac events admissions though they showed large RR values for cold effect on the second day after exposure and on the present day for heat effect. This might be inconsistent with what past studies found with their data. One possible explanation is that widespread use of central heating system in Beijing, a typical northern city in China, have improved the adaptation to cold ambient temperature in winter and usage of air conditioner in summer could relieve the hot stress in summer^39,40^. Besides, the ethnicity of the study participants, the climatic conditions of study area and the environmental background may contribute to the differences in the results^41^. These discrepant findings indicate that further large-scale epidemiological studies and subsequent meta-analyses are thus needed in different climate zones to determine these relationships with certainty.

In addition, the present study observed larger size of cold effect estimates in those over 65 years old. Several previous studies reported similar findings that extremely low temperature was more likely to induce an acute cardiovascular event in susceptible individuals such as the elderly^18,26,41–44^. A possible explanation for this increased susceptibility in the elderly relates to a reduced thermoregulation, together with a decreased capacity in perception of changes in body temperature^42,45^.

A novelty of this study is the comparative investigation on the relationship between AT and specific subtypes of time-sensitive acute cardiac events and ACS (STEMI and NSTEACS) whilst most previous studies have examined emergency department visits in general or a single disease (e.g. STEMI, AMI). There is no agreed conclusion about how effects of temperature may vary on different subtypes of ACS. In the present study, admissions due to STEMI was only affected by extreme high AT while NSTE-ACS admission was found to be only affected by extreme low AT. Contrary to the association of AT and STEMI in the present study, a study conducted in two large Italian urban areas reported that colder days were the days of higher risk of STEMI^6^. However, a research from Japan found that both lower mean temperature and increased maximum temperature from the previous day were independently associated with the STEMI occurrence throughout the year^46^. With regard to the difference between the impact of temperature on STEMI and NSTEMI, a study comparing the seasonal variation of STEMI and NSTEMI in Israel demonstrated significant increases in STEMI cases during the winter months whereas no statistically significant differences in the seasonal variation were found for NSTEMI^47^. Furthermore, seasonal variations in the incidence of ACS could reflect differences in the underlying pathobiology^48^. Although our results are not the same, these findings do support the concept that the underlying pathophysiology of STEMI is different than that of remaining types of ACS. And inconsistency in these findings merits further investigation focusing especially on the comparative investigation of differences in relationship between temperature and subtypes of ACS. Also, further larger scale original studies or systematic reviews concerning the causal effect of short-term exposure to extreme temperature on subtypes of ACS risk are needed to resolve this discrepancy.

Another novelty in the present study is that we employ a biometeorological index AT as exposure measurement to evaluate the effects of temperature on acute cardiac events, rather than daily mean, minimum or maximum temperature. Human body is exposed to a set of atmospheric parameters simultaneously and AT could capture the reaction of the human body to the combined conditions of the thermal environment. It has been used in some studies exploring the impact of ambient temperature on mental and behavioral health^24,27,28^ and identified as the main predictor of heat-related mortality^49^. To date, however, the relationship between AT and admissions due to acute cardiac events or ACS has not been reported in literature. Compared with previous studies using daily mean temperature as exposure variable, AT can provide a measure of the combined effect of environmental variables on human health and wellbeing, and can estimate the human perception of temperature^50^.

There are several potential limitations of this study. First, this is a retrospective ecological study and the ecologic fallacy cannot be ruled out. While causation cannot be directly inferred from our work, ATs were all individually capable of predicting risk well before the adverse event. Second, the single-center and relatively small sample size of this study may limit the generalization of these findings to the whole population. And thus, the association of AT with acute cardiac events and whether the effect varies by subtypes of ACS should be explored in future studies. Third, meteorological data were extracted from fixed monitoring sites rather than individual exposure measures, which may lead to measurement errors. However, we linked the daily emergency admissions of each hospital to the daily meteorological measures in the corresponding district to minimize the misclassification. Fourth, seasonal influenza was associated with an increase in MI-associated deaths and hospitalizations in previous studies^51^. However, our analysis failed to control for influenza as a possible confounder due to the unavailability of data, and the results may show an overestimate of the effect in cold seasons.

## Conclusion

Both high and low extreme ATs were found to be risk factors for hospital admissions due to acute cardiac events and ACS. The study also observed detrimental effect of high extreme AT on STEMI and low extreme AT on NSTE-ACS. These findings may help better understand the relationship between temperature and the onset of acute cardiac events, and will contribute to developing public health intervention measures, as well as planning of clinical service and emergency resources to reduce mortality caused by untimely emergency treatment in ACS patients. Further multi-center studies aiming to differentiate the effect of non-optimal temperature on ACS subtypes are warranted.

## Supplementary Information

Supplementary Information.

## Data Availability

The associated study protocol and data collection tools will be made available upon request from the corresponding author. Datasets are partly available from the corresponding author upon reasonable request after the completion of primary analyses and results dissemination.
