# Supplementary material for: Associations of apparent temperature with acute cardiac events and subtypes of acute coronary syndromes in Beijing, China
Source: Sci Rep. 2021 Jul 27;11:15229. doi: 10.1038/s41598-021-94738-9 (PMC8316341; doi:10.1038/s41598-021-94738-9)
Supplement: Supplementary file 1 — Supplementary Information. [file 41598_2021_94738_MOESM1_ESM.pdf]

Associations of apparent temperature with acute cardiac events and subtypes of acute coronary syndromes in Beijing, China

Na Li, M.S.[1,2], Junxiong Ma, M.S.[1,2], Fangjing Liu, M.S.[1,2], Yan Zhang, M.D.[3], Pengkun Ma, Ph.D[4], Yinzi Jin, Ph.D[1,2*], Zhi-Jie Zheng, M.D., Ph.D[1,2#]

1 Department of Global Health, School of Public Health, Peking University, Beijing, China

2 Institute for Global Health and Development, Peking University, Beijing, China

3 Institute of Cardiovascular Disease, Peking University First Hospital, Beijing, China

4 Institute of Urban Meteorology, Chinese Meteorological Administration, Beijing, China

[*]**Correspondence to**: Yinzi Jin, PhD, Department of Global Health, Peking University School of Public Health, 38 Xue Yuan Road, Haidian District, Beijing 100191, China

E-mail: yzjin@bjmu.edu.cn

Fax number: +8610-82802144

Telephone number: +86-15001081989

[#]**Senior author**: Zhi-Jie Zheng, MD, PhD, Department of Global Health, School of Public Health, Peking University, 38 Xue Yuan Road, Haidian District, Beijing 100191, China. E-mail: zhengzj@bjmu.edu.cn

Supplementary Information

Table S1 The single day effects of high AT for admissions due to acute cardiac events cross different lag day(s) in age-specific groups, with the reference of the Minimum Admission Apparent Temperature (MAAT) of each group.

	Cold Effect		Hot Effect	
	below 65 years old	aged 65 and above	below 65 years	aged 65 and above
lag0	0.630 (0.413,0.961)	0.646 (0.324,1.29)	1.704 (1.195,2.429)	0.975 (0.912,1.043)
lag1	1.410 (1.085,1.833)	1.718 (1.151,2.564)	0.827 (0.667,1.026)	1.008 (0.959,1.061)
lag2	1.192 (0.992,1.433)	1.460 (1.097,1.943)	0.806 (0.691,0.939)	1.005 (0.971,1.04)
lag3	0.882 (0.779,1.000)	1.028 (0.84,1.258)	0.934 (0.833,1.047)	0.995 (0.969,1.021)
lag4	0.822 (0.717,0.943)	0.915 (0.735,1.138)	1.001 (0.887,1.13)	0.993 (0.966,1.021)
lag5	0.876 (0.791,0.97)	0.937 (0.795,1.103)	1.021 (0.933,1.117)	0.996 (0.976,1.015)
lag6	0.964 (0.894,1.04)	0.998 (0.883,1.129)	1.021 (0.954,1.092)	0.999 (0.986,1.013)
lag7	1.034 (0.954,1.121)	1.045 (0.918,1.19)	1.020 (0.95,1.095)	1.002 (0.987,1.018)
lag8	1.080 (0.990,1.179)	1.072 (0.932,1.234)	1.020 (0.945,1.101)	1.004 (0.987,1.022)
lag9	1.103 (1.012,1.202)	1.081 (0.943,1.24)	1.021 (0.948,1.099)	1.005 (0.989,1.022)
lag10	1.105 (1.023,1.193)	1.075 (0.951,1.216)	1.021 (0.956,1.091)	1.006 (0.992,1.02)
lag11	1.090 (1.016,1.169)	1.057 (0.945,1.183)	1.023 (0.962,1.087)	1.006 (0.995,1.017)
lag12	1.063 (0.981,1.152)	1.03 (0.907,1.171)	1.024 (0.953,1.1)	1.006 (0.993,1.019)
lag13	1.028 (0.921,1.149)	0.999 (0.839,1.189)	1.026 (0.929,1.133)	1.005 (0.986,1.025)
lag14	0.992 (0.851,1.155)	0.965 (0.76,1.226)	1.028 (0.896,1.179)	1.004 (0.976,1.033)

Notes: MAAT for <65 group and ≥65 group was 20°C and 33°C, respectively.

Table S2 The single day effects of high AT for admissions due to acute cardiac events cross different lag day(s) in gender-specific groups, with the reference of the Minimum Admission Apparent Temperature (MAAT) of each group.

	Cold Effect		Hot Effect	
	Male	Female	Male	Female
lag0	0.588 (0.39,0.889)	0.495 (0.222,1.103)	1.326 (0.976,1.801)	1.038 (0.959,1.123)
lag1	1.499 (1.165,1.93)	1.785 (1.126,2.831)	0.883 (0.728,1.070)	0.999 (0.943,1.06)
lag2	1.225 (1.025,1.463)	1.579 (1.135,2.195)	0.858 (0.749,0.983)	0.983 (0.944,1.023)
lag3	0.862 (0.763,0.973)	1.072 (0.85,1.352)	0.930 (0.839,1.031)	0.979 (0.95,1.009)
lag4	0.798 (0.698,0.911)	0.937 (0.729,1.205)	0.982 (0.88,1.095)	0.981 (0.95,1.013)
lag5	0.865 (0.783,0.955)	0.954 (0.79,1.152)	1.015 (0.937,1.101)	0.987 (0.965,1.01)
lag6	0.973 (0.904,1.047)	1.016 (0.882,1.169)	1.036 (0.975,1.101)	0.993 (0.977,1.009)
lag7	1.058 (0.978,1.144)	1.061 (0.914,1.232)	1.047 (0.982,1.117)	0.998 (0.981,1.016)
lag8	1.110 (1.02,1.209)	1.087 (0.925,1.276)	1.051 (0.981,1.127)	1.002 (0.983,1.022)
lag9	1.131 (1.04,1.229)	1.093 (0.934,1.279)	1.049 (0.980,1.122)	1.005 (0.986,1.024)
lag10	1.123 (1.042,1.211)	1.084 (0.941,1.249)	1.040 (0.980,1.104)	1.007 (0.991,1.024)
lag11	1.094 (1.021,1.171)	1.062 (0.933,1.209)	1.028 (0.973,1.086)	1.009 (0.996,1.022)
lag12	1.049 (0.97,1.135)	1.032 (0.890,1.196)	1.012 (0.949,1.079)	1.010 (0.995,1.025)
lag13	0.997 (0.895,1.11)	0.997 (0.815,1.219)	0.994 (0.91,1.087)	1.011 (0.988,1.034)
lag14	0.942 (0.813,1.092)	0.960 (0.729,1.264)	0.976 (0.863,1.104)	1.011 (0.978,1.046)

Notes: MAAT for male and female group was 33°C and 22°C, respectively.
